# Clonal Spread of pESI-Positive Multidrug-Resistant ST32 Salmonella enterica Serovar Infantis Isolates among Broilers and Humans in Slovenia

**DOI:** 10.1128/spectrum.02481-22

**Published:** 2022-10-17

**Authors:** Bojan Papić, Darja Kušar, Jasna Mićunović, Mateja Pirš, Matjaž Ocepek, Jana Avberšek

**Affiliations:** a Institute of Microbiology and Parasitology, Veterinary Faculty, University of Ljubljana, Ljubljana, Slovenia; b Institute of Microbiology and Immunology, Faculty of Medicine, University of Ljubljana, Ljubljana, Slovenia; Agriculture and Agriculture-Food Canada

**Keywords:** *Salmonella* Infantis, ST32, pESI, multidrug resistance, broilers, humans

## Abstract

Salmonella enterica subsp. *enterica* serovar Infantis is the most prevalent serovar found in broilers and broiler meat and is among the top five serovars responsible for human infections in Europe. In 2008, a multidrug-resistant *S*. Infantis isolate emerged in Israel with a mosaic megaplasmid named pESI, associated with increased virulence, biofilm formation, and multidrug resistance. Since then, *S.* Infantis clones with pESI-like plasmids have been reported worldwide, replacing pESI-free clones. Here, we typed 161 *S*. Infantis isolates of poultry (*n *= 133) and human clinical (*n *= 28) origin using whole-genome sequencing. The isolates were collected between 2007 and 2021. In addition, we performed PacBio/Illumina sequencing for two representative pESI-like plasmids and compared them with publicly available sequences. All isolates belonged to sequence type 32 (ST32), except for one isolate that represented a novel single-locus variant of ST32. Core genome MLST (cgMLST) analysis revealed 14 clusters of genetically closely related isolates, of which four suggested broiler-to-human transmission of *S*. Infantis. pESI-like plasmids were present in 148/161 (91.9%) isolates; all were highly similar to the publicly available pESI-like sequences but lacked extended-spectrum beta-lactamase (ESBL) genes. PacBio/Illumina hybrid assembly allowed the reconstruction of two novel complete pESI variants. The present study revealed that the multidrug-resistant, pESI-positive *S.* Infantis clone became the predominant *S*. Infantis clone in Slovenian broilers and humans during the last decade. Continued surveillance of resistant *S*. Infantis clones along the food chain is needed to guide public health efforts.

**IMPORTANCE** Salmonella Infantis clones with pESI-like plasmids harboring several virulence and resistance genes have been reported worldwide. In the present study, we compared the population structure of 161 Salmonella Infantis isolates obtained from humans and broilers in Slovenia from 2007 to 2021. Whole-genome sequencing showed that most human isolates clustered apart from broiler isolates, suggesting an alternative source of infection. Most isolates were multidrug resistant due to the presence of pESI-like plasmids, of which two variants (pS89 and pS19) were fully reconstructed using long-read sequencing. Both exhibited high similarity with the original Israeli pESI plasmid and German p2747 plasmid. The prototype plasmid pS89 harbored the typical pESI-associated resistance genes *aadA1*, *qacE*Δ*1*, *sul1*, and *tet*(A), which were absent in the truncated plasmid pS19.

## INTRODUCTION

Salmonella enterica subsp. *enterica* serovar Infantis is the fourth most common Salmonella serovar causing human salmonellosis in Europe (1). It is the most prevalent serovar in broilers and can thus spread along the food chain (1). In 2014, multidrug-resistant (MDR) *S.* Infantis with a megaplasmid known as plasmid of emerging *S.* Infantis (pESI) was first reported for Israeli isolate 119944 from 2008 (2). Since then, *S.* Infantis clones with pESI-like plasmids have been reported worldwide (3–7).

These plasmids exhibit a mosaic structure and are associated with increased virulence and multidrug resistance, contributing to the success of pESI-positive *S*. Infantis clones observed over the past decade (2, 4, 8). The pESI-borne antimicrobial resistance genes (ARGs) are encoded in mobile genetic modules integrated into pESI resistance region 1 or 2 (4, 9). Recently, pESI-like plasmids with extended spectrum beta-lactamase (ESBL) genes have been reported worldwide (3, 4, 7, 9–13). Due to the large size of pESI-like plasmids, which can reach 323 kb (4), short-read next-generation sequencing needs to be complemented by long-read sequencing technologies. However, few studies have used the latter to reconstruct complete, gap-free sequences of pESI (4, 9, 10, 14, 15).

Here, we typed 161 *S*. Infantis isolates from poultry and humans using whole-genome sequencing (WGS). We performed a comprehensive genetic characterization of isolates to assess their epidemiology, population structure, and resistance in the Slovenian *S*. Infantis population. In addition, we used a state-of-the-art hybrid assembly to obtain complete sequences of two representative pESI-like plasmids and compared them with publicly available sequences.

## RESULTS

### WGS analysis.

Multilocus sequence typing (MLST) revealed that all isolates except one (*n *= 160) were of sequence type 32 (ST32); isolate S113 was a single locus variant of ST32 (*thrA*). *In silico* serotyping classified 158/161 isolates as *S*. Infantis (7:r:1,5), whereas three isolates (S20, S165, and S202) lacked the O antigen (−:r:1,5) but had been previously identified as *S*. Infantis by conventional slide agglutination (data not shown).

Presence/absence analysis of genes characteristic of pESI revealed that 148/161 (91.9%) isolates were pESI^+^ (see Tables S1 and S2 in the supplemental material). The first pESI^+^ isolates were observed in 2010 and have since completely replaced the pESI-negative isolates in broiler farms. The occurrence of pESI^+^ isolates was significantly higher in broiler isolates (128/132) than human isolates (20/28) (Fisher’s exact test, *P = *0.0001). IncI1 plasmid MLST (pMLST) classified pESI-like plasmids into five types (Table S1); most pESI^+^ isolates (133/148; 89.9%) had the following allele profile: *ardA*_2, *trbA*_21, *sogS*_9, *pilL*_3.

**FIG 1:**
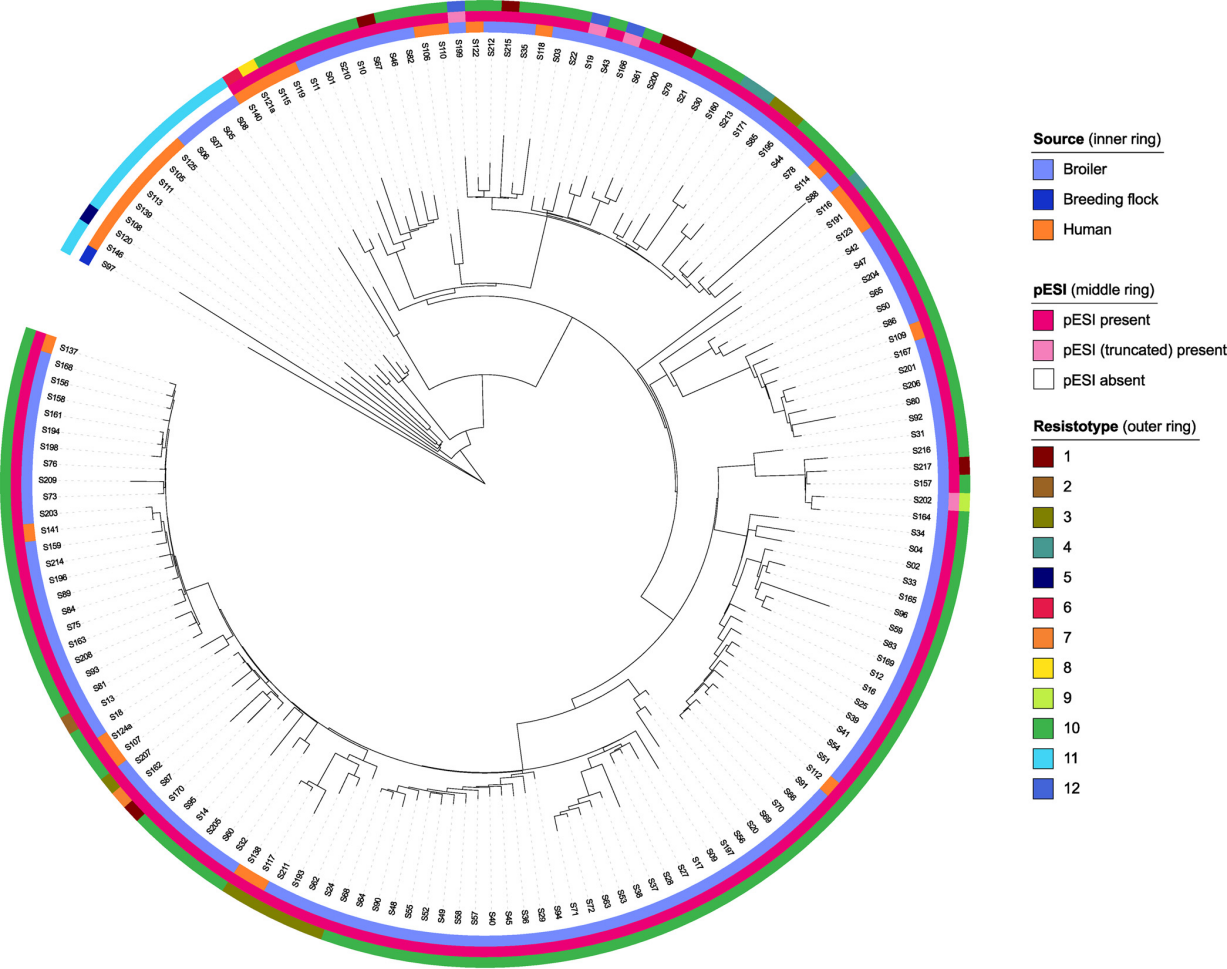
cgMLST tree of 161 Salmonella Infantis isolates. Isolation source, presence of pESI, and resistotype are provided. Resistotypes are numbered as follows: 1, *tet*(A), *aac(6*′)*-Iaa*, *aadA1*, *sul1*, *parC* (T57S), *gyrA* (S83Y), *qnrS1*, *bla*_TEM-1B_, *nfsA* (NS159); 2, *tet*(A), *aac(6*′)*-Iaa*, *aadA1*, *sul1*, *parC* (T57S), *gyrA* (S83Y), *bla*_TEM-1B_; 3, *tet*(A), *aac(6*′)*-Iaa*, *aadA1*, *sul1*, *parC* (T57S), *gyrA* (S83Y), *bla*_TEM-1C_, *nfsA* (NS159); 4, *tet*(A), *aac(6*′)*-Iaa*, *aadA1*, *sul1*, *parC* (T57S), *gyrA* (S83Y), *bla*_TEM-1D_, *nfsA* (NS159); 5, *tet*(A), *aac(6*′)*-Iaa*, *ant(2*′′)*-Ia*, *sul1*, *dfrA1*, *cmlA1*, *parC* (T57S), *gyrA* (D87Y), *bla*_TEM-1B_; 6, *tet*(A), *aac(6*′)*-Iaa*, *aadA1*, *aph(3*′′)*-Ia*, *aph(3*′′)*-Ib*, *aph(6)-Id*, *sul1*, *sul2*, *dfrA14*, *floR*, *parC* (T57S), *gyrA* (S83Y), *bla*_TEM-1A_, *nfsA* (NS159); 7, *tet*(A), *aac(6*′)*-Iaa*, *aadA1*, *aadA2*, *sul1*, *sul3*, *dfrA8*, *cmlA1*, *parC* (T57S), *gyrA* (S83Y), *bla*_TEM-1B_, *nfsA* (NS159); 8, *tet*(A), *aac(6*′)*-Iaa*, *aadA1*, *sul1*, *dfrA14*, *parC* (T57S), *gyrA* (S83Y), *nfsA* (NS159); 9, *aac(6*′)*-Iaa*, *aadA1*, *sul2*, *dfrA1*, *parC* (T57S), *gyrA* (S83Y), *bla*_TEM-1B_, *nfsA* (NS159); 10, *tet*(A), *aac(6*′)*-Iaa*, *aadA1*, *sul1*, *parC* (T57S), *gyrA* (S83Y), *nfsA* (NS159); 11, *aac(6*′)*-Iaa*, *parC* (T57S); 12, *aac(6*′)*-Iaa*, *parC* (T57S), *gyrA* (S83Y), *nfsA* (NS159).

The core genome MLST (cgMLST) tree showed that the pESI^−^ isolates clustered to the exclusion of pESI^+^ isolates (Fig. 1). The latter formed a single monophyletic clade and were genetically fairly homogeneous, with a median intracluster pairwise distance of 83 alleles (range, 2 to 184). pESI^+^ isolates differed from pESI^−^ isolates in ≥102 alleles. With regard to pESI^−^ isolates, the broiler isolates were more genetically homogenous and clustered apart from the human and breeding flock isolates. No evident clustering was observed with respect to isolation source or pESI variant (prototype versus truncated) (Fig. 1). On the contrary, general clustering of isolates according to the food business operator (FBO) was observed with few exceptions (see Fig. S1). Comparison of the isolates originating from the same farms revealed the presence of genetically closely related (≤30 allele differences) isolates in the farm; however, reintroduction of additional genetically distanced strains was also observed over time (data not shown).

**FIG 2:**
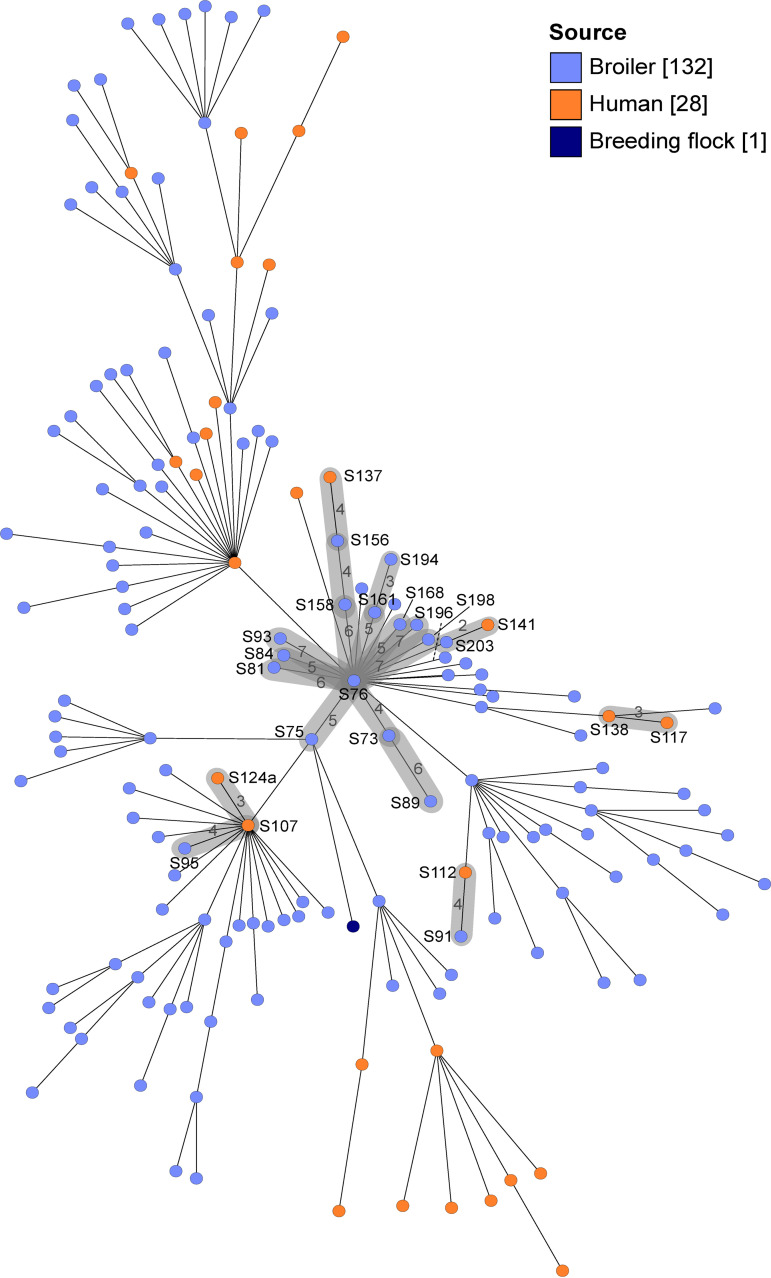
cgMLST minimum spanning tree of 161 Salmonella Infantis isolates. Isolates are colored according to the isolation source. Clusters of closely related (≤7 allele differences) isolates are highlighted in gray; isolates S117 and S138 originated from the same patient. Broiler-to-broiler transmission clusters are not shown. Numbers on the connecting lines indicate the number of allele differences. Other isolate names and distances are omitted for clarity.

Four presumable broiler-to-human transmission clusters were detected, each comprising at least two isolates with ≤7 allele differences obtained over a limited time span (highlighted in Fig. 2). Additionally, one cluster included only two human isolates (S117 and S138), which originated from the same patient. Most (21/28) human isolates did not cluster with broiler isolates. Nine clusters containing only broiler isolates were observed but are not shown in Fig. 2 because of their *de facto* epidemiological linkage (Table S1). Six of nine broiler clusters were limited to a single farm and contained strains that presumably persisted up to 2 years. Isolates of cluster 1 (FBO 3) originated from eight farms and were isolated in 2017 to 2020 (Table S1), whereas isolates from FBOs 1 and 2 were more genetically diverse (Fig. S1).

The presence/absence profiles of ARGs and resistance-associated mutations are shown in Table S3; their genetic background is shown in Table S4 and summarized in Table 1. The isolates exhibited 12 different resistotypes, and 145/161 (90.1%) isolates were MDR (Fig. 1; Table S3). A total of 20 different acquired ARGs conferring resistance to six antimicrobial groups were observed, in addition to the *nfsA* mutation conferring nitrofurantoin resistance.

**TABLE 1:** Resistance plasmids identified *in silico* in the studied Salmonella Infantis isolates^*a*^

Incompatibility group	Best BlastN hit(s)	Mobility group	No. of isolates (% prevalence)^*b*^	ARGs^*c*^
IncFIB (pESI)	CP047882.1	Conjugative	148 (91.9)	*tet*(A) (144/148), *aadA1* (143/148), *aph(3*′)*-Ia* (1/148), *sul1* (144/148), *dfrA14* (2/148), *floR* (1/148), *bla*_TEM-1C_ (8/148)
IncX1	CP023660.1, CP088733.1, CP024467.1, KF362122.2, MH121702.1	Conjugative	8 (5.0)	*qnrS1* (5/8), *bla*_TEM-1D_ (2/8), *bla*_TEM-1B_ (6/8)
NA	CP054381.1, CP035844.1, MN915013.1, CP038299.1	Nonmobilizable	3 (1.9)	*tet*(A) (1/3), *aadA2b* (1/3), *ant(2*′′)*-Ia* (1/3), *sul3* (1/3), *dfrA1* (1/3), *dfrA8* (1/3), *cmlA1* (2/3), *bla*_TEM-1B_ (2/3)
IncIγ/K1	MK070495.1	Conjugative	1 (0.6)	*aadA1* (1/1), *sul2* (1/1), *dfrA1* (1/1), *bla*_TEM-1B_ (1/1)
IncN	CP025233.1	Conjugative	1 (0.6)	*aph(6)-Id* (1/1), *aph(3*′′)*-Ib* (1/1), *sul2* (1/1)
IncX4	MW390518.1	Conjugative	1 (0.6)	*bla*_TEM-1C_ (1/1)

a For a detailed description of the genetic background of each ARG, see Table S4. NA, not assigned.

b *n *= 161.

c Values in parentheses are numbers of ARG-positive isolates/plasmid-positive isolates.

Regarding pESI-encoded ARGs, 144/148 pESI^+^ isolates simultaneously harbored *aadA1* (streptomycin resistance), *tet*(A) (tetracycline resistance), and *sul1* (sulfonamide resistance) (Table 1; Tables S2 and S3). Seven different ARGs were encoded by pESI-like plasmids (Table 1; Table S4). Four pESI^+^ isolates had no ARGs in any of the resistance regions and thus harbored a presumed truncated pESI variant (Tables S1, S2, and S4). All pESI^+^ isolates harbored a chromosomal point mutation in *gyrA* (S83Y) leading to fluoroquinolone resistance.

WGS analysis revealed that most of the acquired ARGs were plasmid borne. In addition to *tet*(A), *aadA1*, and *sul1*, carried in resistance region 2, pESI-like plasmids also harbored *bla*_TEM-1C_ (8/148 isolates) in resistance region 2 and *aph(3*′)*-Ia* (1/148 isolates), *dfrA14* (2/148 isolates), and *floR* (1/148 isolates) in resistance region 1. In addition to pESI-like plasmids, nonmobilizable plasmids and conjugative plasmids of the incompatibility groups IncX1, IncN, IncIγ/K1, and IncX4 were also observed (Table 1).

All Slovenian pESI-like plasmids except the four truncated variants carried genes involved in resistance to quaternary ammonium compounds (*qacE*Δ*1*) and mercury (*mer*), both in resistance region 2, whereas none had *ars* genes, involved in arsenic tolerance. All pESI-like plasmids harbored the virulence- and fitness-associated traits located in the conserved pESI backbone, such as the yersiniabactin iron acquisition system (*ybt*) and two chaperone-usher fimbrial gene clusters (*fae* and *ipf*) (Table S2).

### Comparative analysis of pESI-like plasmids.

**FIG 3:**
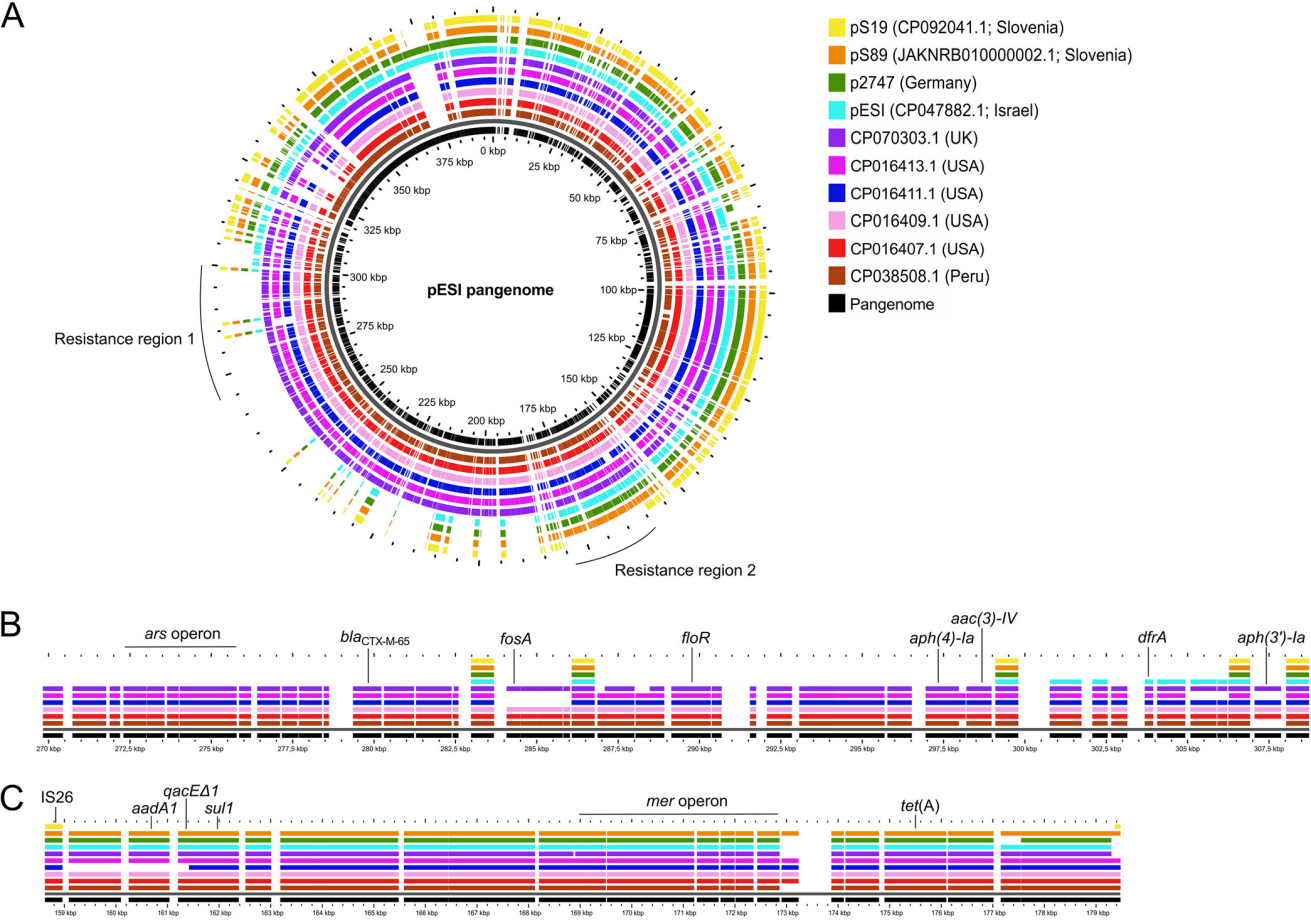
Pangenome analysis of Slovenian pESI-like plasmids pS19 and pS89 compared with the original Israeli pESI and other pESI-like plasmids. (A) Pangenome analysis; (B) resistance region 1; (C) resistance region 2. Selected genes are highlighted in panels A and B. For isolate metadata, see Table S6.

The complete, gap-free pESI-like sequence of the prototype Slovenian pESI-like plasmid pS89 confirmed the integration of the resistance genes *aadA1*, *qacE*Δ*1*, *tet*(A), and *sul1* in resistance region 2 (Fig. 3). The complete, gap-free sequence of pS19 confirmed that it lacked all ARGs in resistance regions 1 and 2 (Fig. 3).

The comparative analysis of pESI sequences revealed that pS19 and pS89 are genetically closely related (Fig. 3); this was confirmed by high average nucleotide identity based on the MUMmer algorithm (ANIm) (99.97% identity over a coverage of 89.97%) (Table S5). The Slovenian pESI-like plasmids pS89 and pS19 were most similar to the German p2747 and the original Israeli pESI (Fig. 3), with a minimum identity of 99.95% (Table S6). Plasmid pS89 harbored the same resistance genes as plasmid p2747, which both lacked *dfrA14*, in contrast to the original pESI (Fig. 3).

## DISCUSSION

In the present study, we used WGS for the first time to elucidate the population structure of *S*. Infantis in Slovenia. Previously, we had observed an increasing rate of MDR *S*. Infantis in Slovenian broiler farms (16). We show here that the MDR pESI^+^ ST32 *S*. Infantis clone became the predominant clone in Slovenian broiler farms over the last decade. Most clinical isolates from humans were also of ST32 and harbored pESI. The success of *S*. Infantis ST32 can be explained, at least in part, by the presence of the virulence and resistance megaplasmid pESI (15).

We reconstructed two Slovenian pESI-like plasmids using state-of-the-art hybrid assembly, increasing the number of pESI sequences available in public databases. Their comparison with publicly available pESI-like plasmids from different geographical regions confirmed the highly mosaic structure with two variable resistance regions integrated into a conserved pESI backbone (4, 9, 10). Whereas the prototype variant pS89 had the resistance genes *aadA1*, *tet*(A), and *sul1* (all in resistance region 2), the truncated variant pS19 lacked all ARGs, *qacE*Δ*1*, and the *mer* operon. Therefore, the isolates with truncated pESI were resistant only to fluoroquinolones and nitrofurantoin and were not MDR. Interestingly, the broiler isolate S202 harbored a truncated pESI but was MDR due to ARGs present on an additional IncIγ/K1 plasmid. A similar case was observed with the human isolate S120 (pESI^−^), which harbored ARGs on a nonmobilizable plasmid conferring resistance to five antimicrobial groups. With respect to the presence of additional resistance plasmids, no evident clustering of isolates was observed, suggesting their horizontal transfer between different *S.* Infantis clones.

In this study, seven pESI-encoded ARGs conferring resistance to five antimicrobial classes (tetracyclines, aminoglycosides, folate pathway antagonists, phenicols, and penicillins) were observed with variable presence; ESBL determinants and colistin resistance genes were not observed in any of the studied isolates. With regard to fluoroquinolone resistance, the S83Y mutation in *gyrA* was observed in all (*n *= 148) pESI^+^ isolates, the D87Y mutation in one pESI^−^ human isolate (S120), and *qnrS1* in 6/148 pESI^+^ isolates. Fluoroquinolones and third-generation cephalosporins are among the few treatment options for severe human salmonellosis cases (17). Therefore, treatment could be further complicated by integration of additional resistance genes into pESI or acquisition of additional resistance plasmids, both of which were observed here. High genome plasticity of pESI-like plasmids may facilitate evolutionary adaptation of *S*. Infantis to different environments and selection pressures, as previously suggested (2, 8).

Four isolates with the truncated pESI variant (including S19) were scattered throughout the pESI-positive clade in the cgMLST tree. Therefore, the truncated variant pS19 cannot be regarded as parental to the prototype plasmid pS89, but rather, it can be speculated that such variants arose independently multiple times. Truncated pESI variants without ARGs have also been reported previously (10, 18).

The predominant pMLST type of pESI observed in this study is associated with group 2 pESI, as defined by Bogomazova et al. (6), which has already been described as a carrier of the ESBL determinant *bla*_CTX-M-1_. In addition, the presence of certain ARGs in the resistance region 2 [*aph(3*′)*-Ia*, *dfrA14*, and *floR*] suggests that ESBL determinants could be easily integrated into the prototype Slovenian pESI-like plasmid.

pESI^+^ isolates predominated in both broilers and humans, although their prevalence was significantly higher in broilers. This suggests a stable integration of the pESI^+^ clone into both populations during the last decade. Here, four presumable broiler-to-human transmission clusters were detected, which has been rarely confirmed using WGS (4, 11). Nevertheless, most human isolates did not cluster with the broiler isolates, suggesting that Slovenian broilers cannot be regarded as an important source of infection for humans. However, future studies should also include WGS typing of isolates from imported meat. Another limitation of this study was the lack of data on human patient travel history, which would assess the impact of imported human cases. Due to the mosaic structure of pESI-like plasmids, continuous surveillance of antimicrobial resistance in *S*. Infantis is essential. Since conjugative transfer of pESI from *S*. Infantis to Escherichia coli has also been demonstrated both *in vitro* and *in vivo* (2, 8), detection of pESI in non-*S.* Infantis hosts should not be neglected.

We showed that the MDR pESI^+^ *S*. Infantis clone is predominant in broiler farms in Slovenia and has become increasingly common since 2010. The high genetic plasticity of pESI-like plasmids represents a threat for the introduction of ESBL genes into the Slovenian *S.* Infantis population. The presumable transmission clusters observed highlight the zoonotic potential of *S.* Infantis with limited treatment options.

## MATERIALS AND METHODS

### Isolate panel.

The analyzed *S*. Infantis panel (*n *= 161) consisted of isolates from broiler feces (*n *= 132), one isolate from a breeding flock, and 28 clinical isolates from humans (fecal, urine, and blood samples) from 2007 to 2021 (Table S1). The poultry isolates were selected from the isolate collection of the Institute of Microbiology and Parasitology, Veterinary Faculty. They originated from three large-scale FBOs in Slovenia, with farms distributed throughout the country, and eight independent farmers. The human isolates originated from sporadic human salmonellosis cases and represented the complete *S.* Infantis collection of the Institute of Microbiology and Immunology, Faculty of Medicine. For isolate metadata, see Table S1.

### Illumina sequencing.

*S.* Infantis isolates were grown overnight on blood agar plates, and genomic DNA was extracted using a DNeasy blood and tissue kit (Qiagen, Hilden, Germany). All isolates underwent Illumina paired-end (2 × 150 bp) sequencing to a minimum coverage of 100×; details about the library preparation kits and sequencing platforms used in this study are provided in Table S1. The quality of the raw reads was assessed using FastQC (https://www.bioinformatics.babraham.ac.uk/projects/fastqc/). Reads were assembled with Shovill v1.0.9 (https://github.com/tseemann/shovill) using SPAdes v3.13.1. The quality of the assembly was assessed using Quast (19); the following quality parameters were used: (i) *N*_50_ > 20,000, (ii) total number of contigs longer than 500 bp < 100, and (iii) total assembly size between 4.1 and 5.5 Mb.

### PacBio sequencing.

Based on the preliminary gene content analysis of Illumina data, two strains (S19 and S89) were selected for PacBio long-read sequencing to reconstruct the complete sequences of pESI-like plasmids. Strain S89 harbored the Slovenian prototype pESI-like plasmid pS89 with the resistance genes *sul1*, *aadA1*, and *tet*(A). Strain S19 harbored its truncated variant pS19 with no ARGs. For this purpose, plasmid DNA was extracted from 500 mL of overnight culture grown in buffered peptone water using a NucleoBond Xtra midikit (Macherey-Nagel, Düren, Germany). SMRTbell libraries were sequenced on a PacBio Sequel platform. Hybrid assembly of Illumina and PacBio reads was performed using Unicycler v0.4.8 (20) run in normal mode.

### WGS analysis.

*In silico* 7-gene MLST was performed using the Sequence query tool implemented in the PubMLST Salmonella database (21). pMLST of pESI-like plasmids was performed using pMLST v2.0 (22) according to the IncI1 pMLST scheme. *In silico* serotyping was performed using SeqSero2 v1.1.0 (23).

cgMLST allele calling was performed with chewBBACA (24) using the Prodigal training file for Salmonella enterica (https://github.com/B-UMMI/chewBBACA/tree/master/CHEWBBACA/prodigal_training_files). The initial *ad hoc* whole-genome MLST scheme consisted of 5,399 loci; after removal of 1,448 noncore loci, the final cgMLST scheme consisted of 3,951 loci. The circular cgMLST tree was created using the neighbor-joining algorithm implemented in Grapetree (25) and then annotated and visualized using iTOL v6.3 (26). The cgMLST minimum spanning tree was constructed using Grapetree v1.5.0 (25) and the MSTreeV2 algorithm. A single-linkage threshold of seven alleles (27) was used for cluster identification.

Detection of acquired ARGs and antimicrobial resistance-associated chromosomal mutations was performed using ResFinder v4.1 (28) using the cutoff thresholds of 60% coverage and 90% identity. In addition, the assemblies were screened for the presence of a nonsense mutation at position 159 of NfsA (nitrofurantoin resistance) in Geneious Prime v2021.1.1 (Biomatters, Auckland, New Zealand).

### Comparative analysis of pESI-like plasmids.

BLASTn was used to detect the selected virulence- and fitness-associated genes in the conserved region of pESI (Table S2) by applying the cutoff values of 60% coverage and 90% identity. The isolates harboring the conserved pESI genes were defined as pESI^+^.

The reconstructed Slovenian pESI-like plasmids were compared with the publicly available sequences listed in Table S6. Pangenome analysis of pESI-like sequences was performed using GView Server v1.7 (20). MOB-recon integrated into MOB-suite tools v3.0.3 (29) was performed to reconstruct and type the plasmid sequences from draft genomes. The detected plasmid sequences were manually examined using BLASTn. ANIm values were calculated using JSpeciesWS (30).

### Data availability.

All WGS read data (Table S1) and pESI-like plasmid sequences (Table S6) obtained in this study have been deposited in the NCBI Sequence Read Archive (SRA) and nucleotide database, respectively, under BioProject accession number PRJNA803004.
