# Supplementary material for: Clonal Spread of pESI-Positive Multidrug-Resistant ST32 Salmonella enterica Serovar Infantis Isolates among Broilers and Humans in Slovenia
Source: Microbiol Spectr. 2022 Oct 17;10(6):e02481-22. doi: 10.1128/spectrum.02481-22 (PMC9769575; doi:10.1128/spectrum.02481-22)
Supplement: Supplemental file 1 — Fig. S1 and captions for Tables S1 to S6. Download spectrum.02481-22-s0002.pdf, PDF file, 0.2 MB [file spectrum.02481-22-s0002.pdf]

Supplementary materials

Clonal spread of pESI-positive multidrug-resistant ST32 *Salmonella* Infantis among broilers and humans in Slovenia

Bojan Papić, Darja Kušar, Jasna Mićunović, Mateja Pirš, Matjaž Ocepek, Jana Avberšek

This supplementary file includes:

Figure S1 cgMLST tree of 161 *Salmonella* Infantis isolates colored according to the food business operation.

Other supplementary information for this manuscript includes:

Supplementary Tables S1–S6 (separate sheets in the supplementary Excel file named Tables_S1-S6.xlsx):

Table S1 Isolate metadata.

Table S2 Presence/absence profile of pESI-encoded genes.

Table S3 Presence/absence profiles of antimicrobial resistance genes and resistance-associated mutations.

Table S4 Genetic background of antimicrobial resistance.

Table S5 Pairwise ANIm values and alignment coverage (in parenthesis) for the pESI-like plasmids included in this study.

Table S6 List of complete pESI-like plasmid sequences used in this study.

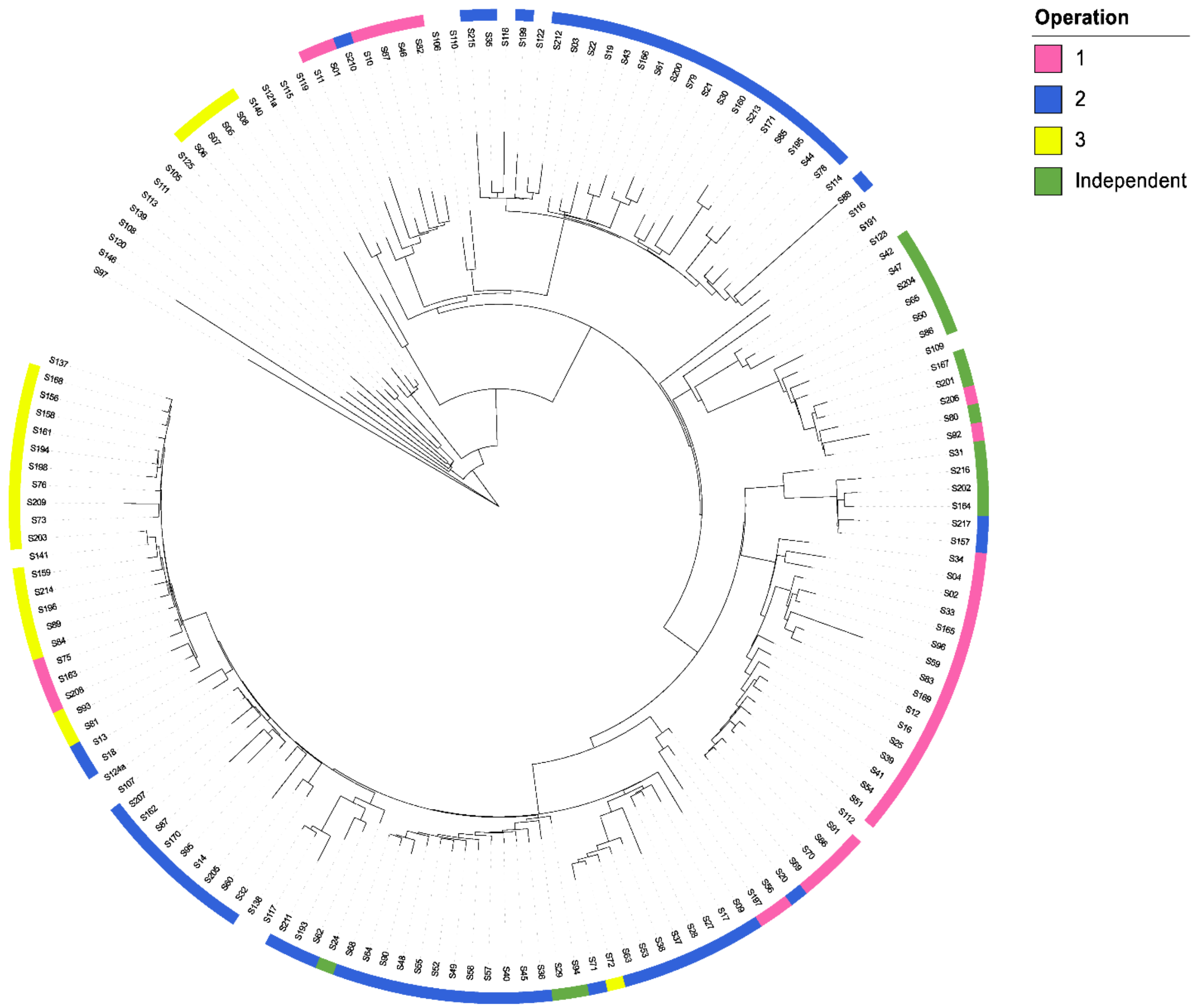

Figure S1 cgMLST tree of 161 *Salmonella* Infantis isolates colored according to the food business operation. Human isolates are denoted in white.
